# Climate-smart rice (*Oryza sativa* L.) genotypes identification using stability analysis, multi-trait selection index, and genotype-environment interaction at different irrigation regimes with adaptation to universal warming

**DOI:** 10.1038/s41598-024-64808-9

**Published:** 2024-06-15

**Authors:** Muhammad Ashraful Habib, Mohammad Golam Azam, Md. Ashraful Haque, Lutful Hassan, Mst. Suhana Khatun, Swati Nayak, Hasan Muhammad Abdullah, Riaz Ullah, Essam A. Ali, Nazmul Hossain, Sezai Ercisli, Umakanta Sarker

**Affiliations:** 1 Department of Genetics and Plant Breeding, Faculty of Agriculture, Bangladesh Agricultural University, Mymensingh, 2202 Bangladesh; 2 Rice Breeding Innovation Platform, International Rice Research Institute, 4031 Los Banos, Laguna Philippines; 3 Pulses Research Centre, Bangladesh Agricultural Research Institute, Ishurdi, Pabna, 6620 Bangladesh; 4 Department of Remote Sensing and GIS, Faculty of Forestry and Environment, Bangabandhu Sheikh Mujibur Rahman Agricultural University, Gazipur, 1706 Bangladesh; 5 Department of Pharmacognosy, College of Pharmacy, King Saud University, 11451 Riyadh, Saudi Arabia; 6 Department of Pharmaceutical Chemistry, College of Pharmacy, King Saud University, 11451 Riyadh, Saudi Arabia; 7 Department of Agronomy, Iowa State University, Ames, IA 50010 USA; 8 Department of Horticulture, Faculty of Agriculture, Ataturk University, 25240 Erzurum, , Turkey; 9 Department of Genetics and Plant Breeding, Faculty of Agriculture, Bangabandhu Sheikh Mujibur Rahman Agricultural University, Gazipur, 1706 Bangladesh

**Keywords:** Rice, MGIDI, AMMI, GGE biplot model, Yield, web-based WIST, Plant breeding, Agricultural genetics

## Abstract

Climate change has brought an alarming situation in the scarcity of fresh water for irrigation due to the present global water crisis, climate variability, drought, increasing demands of water from the industrial sectors, and contamination of water resources. Accurately evaluating the potential of future rice genotypes in large-scale, multi-environment experiments may be challenging. A key component of the accurate assessment is the examination of stability in growth contexts and genotype-environment interaction. Using a split-plot design with three replications, the study was carried out in nine locations with five genotypes under continuous flooding (CF) and alternate wet and dry (AWD) conditions. Utilizing the web-based warehouse inventory search tool (WIST), the water status was determined. To evaluate yield performance for stability and adaptability, AMMI and GGE biplots were used. The genotypes clearly reacted inversely to the various environments, and substantial interactions were identified. Out of all the environments, G3 (BRRI dhan29) had the greatest grain production, whereas G2 (Binadhan-8) had the lowest. The range between the greatest and lowest mean values of rice grain output (4.95 to 4.62 t ha^-1^) was consistent across five distinct rice genotypes. The genotype means varied from 5.03 to 4.73 t ha^-1^ depending on the environment. In AWD, all genotypes out performed in the CF system. With just a little interaction effect, the score was almost zero for several genotypes (E1, E2, E6, and E7 for the AWD technique, and E5, E6, E8, and E9 for the CF method) because they performed better in particular settings. The GGE biplot provided more evidence in support of the AMMI study results. The study's findings made it clear that the AMMI model provides a substantial amount of information when evaluating varietal performance across many environments. Out of the five accessions that were analyzed, one was found to be top-ranking by the multi-trait genotype ideotype distance index, meaning that it may be investigated for validation stability measures. The study's findings provide helpful information on the variety selection for the settings in which BRRI dhan47 and BRRI dhan29, respectively, performed effectively in AWD and CF systems. Plant breeders might use this knowledge to choose newer kinds and to design breeding initiatives. In conclusion, intermittent irrigation could be an effective adaptation technique for simultaneously saving water and mitigating GHG while maintaining high rice grain yields in rice cultivation systems.

## Introduction

Over 90% of the world's rice crop is produced in Asia, where rice (*Oryza sativa* L.) provides the most versatile food source for one-third of the world's population^1^. As the most ancient and consequential crop, rice cultivation not only ensures food security but also generates income and employment possibilities^2^. Globally, 755 million tons of paddy are produced on 162 million hectares of land^3^. Reducing poverty and satisfying the needs of the world's continuously growing population depends on raising the rice grain yield per unit area. 48% of all farm workers in the country are employed in the rice industry, which has 75% of all agricultural land. It provides 70% of the agricultural Gross Domestic Product (GDP) and accounts for 1/6 of the country's total income^4^. Rice is sown all year round in Bangladesh, with the three growth seasons being Boro, Aman, and Aus. According to Shelley et al*.*^5^, it is grown in four different ecosystems: rainfed upland (direct-seeded Aus), rainfed or partly irrigated (transplanted Aus and Aman), and deep-water (broadcast Aman). A large amount of land used to grow rice is being used for other uses. Additionally, a nationwide average of 4.5 t/ha is used to correct the yield performance^6^. Additionally, since man-made events pose a danger to rice breeding, breeders need to be prepared for any challenges that arise from uncertainty. As a result, rice breeders must develop new cultivars with improved yield and stability across a variety of conditions, or that are location-specific.

To combat ongoing floods, water-saving methods have been developed and deployed in paddy cultivation, according to Tuong and Bhuiyan^7^ and Li et al*.*^8^. The adoption of AWD irrigation technology is also growing across various agro-ecological zones and climates^9,10^. Through a series of research, IRRI developed the precise water management method known as AWD, which may save up to 44% of the water used for irrigation in paddy fields throughout the growing season^11^. Tan et al*.*^10^ found that, in comparison to continuous flooding irrigation treatment, there was no discernible yield penalty below AWD and that water productivity increased by 17%. AWD increases production and water usage efficiency while lowering the amount of water needed for seasonal irrigation. In addition, Zhang et al*.*^12^ reported that, in comparison to continuous flooding, AWD might save 35% of the irrigation water while increasing output by 10%. LaHue et al*.*^13^ found no change in yield between the AWD treatments and constant flooding. Li and Barker^14^ noted an increase in water production linked to the use of AWD technology. Howell et al*.*^15^ found that although AWD saved 57% of the irrigation water compared to CF, there was no discernible change in rice yield. While Xue et al*.*^16^ observed a production decline as high as 16%, Humphreys et al*.*^17^ showed a small yield drop under AWD. Even though it has been demonstrated that the AWD practice has benefits in terms of lowering water supply and raising crop productivity, very little research has been done to assess possible water-saving strategies, particularly under varying water application rates in the drought-prone areas of Bangladesh. Plant physiological performances, plant growth parameters, and crop growth rate of rice plants were mostly not significantly affected by water-saving irrigation treatments^18,19^. Plant growth parameters and relative chlorophyll content of rice of all water management treatments showed no significant difference during tillering, active tillering, flowering, and ripening stages. The rice yield achieved through alternate wetting and drying (AWD) and saturated soil irrigation technique does not show a significant difference compared to CF irrigation^20,21^.

In multi-environment trials (METs), a genotype's grain yield performance may vary depending on the environment, suggesting a substantial genotype × environments interaction (GEI). Cross-over interaction refers to the possibility that a high GEI would alter the way genotypes are ranked in various contexts^22^. This makes choosing better genotypes for target environments much more difficult. The better genotype continues to be the highest-performing genotype across all conditions in the absence of GEI^23^. As a result, GEI makes it difficult for plant breeders to identify and select better cultivars, which presents significant hurdles^24^. Optimizing breeding techniques to choose cultivars that are well suited to certain environments requires measuring genetic equivalent inflation^25^. METs find experimental locations that best mimic the target environment in addition to high-yielding and stable genotypes across environments^26,27^.

Numerous techniques are presented for examining the data gathered from METs, and they may be broadly classified into two categories: univariate and multivariate techniques. The additive main effect and multiplicative interaction (AMMI) model has significant relevance when it comes to multivariate approaches. Principal components (PCs) analysis and analysis of variance are essentially combined in the AMMI approach. Analysis of variance is used in the first part of AMMI, or the additive portion^28^; the PCs analysis technique is used in the second part of AMMI, which contains the multiplicative element, to analyze the GEI based on PCs^29^. Actually, the high resolution of GEI and primary effects, together with the explanation of a significant portion of the interaction's sum of squares, account for the model's extensive use^30^.

Plant breeders may find better genotypes in the presence of considerable G × E interaction with the assistance of many statistical techniques currently available for studying genotype-yield adaptation and stability^31^. According to Agyeman et al*.*^32^, AMMI, and genotype plus genotype by environment (GGE), biplot analyses are two often used methods for overcoming problems with multi-environment trial data analysis. The AMMI and GGE biplot models are useful tools for analyzing and offering insights on multi-environment data structures in breeding operations, according to Samonte et al*.*^33^, and Yan et al*.*^34^. Because these two statistical methods (AMMI and GGE) may be used for any two-way data matrix, including the number of genotypes tested in numerous locations and the possibility that the data come from several trials, agricultural researchers are particularly interested in them^35^. Principal component analysis (PCA) and analysis of variance (ANOVA) are used in these investigations^36^. The stability and adaptability of the genotypes have been evaluated using a variety of statistical techniques, such as ANOVA, joint regression analysis, additive main effects and multiplicative interaction (AMMI), and genotype main effect plus genotype-by-environment interaction (GGE) biplots^34,37^. The evaluation of yearly MET data has been done extensively using the useful method known as GGE biplot analysis. The GGE biplot provides a two-dimensional graphical demonstration of the interactions between genotypes and environments in terms of how they discriminate against each other^34^. Consequently, each target set of environments and the AMMI and GGE biplot models made it easier to compare and identify superior genotypes visually^38^. In the context of multiple selection, linear indexes can be used^39^. One fragility of linear selection indexes is the collinearity often observed in the set of assessed traits, which can bias the coefficients of multiple regression, and thus erode selection gains^40^. To overcome this fragility, the multi-trait genotype-ideotype distance index (MGIDI) has been proposed ^41^. The MGIDI was originally designed for selecting genotypes in plant breeding based on information on multiple traits and has been successfully used to select superior genotypes^41,42^. Thus, the current study aimed to ascertain the adaptability of climate-smart promising rice germplasm using AMMI, GGE biplot, and MGIDI analyses for different moisture regimes and to examine their water-saving techniques.

## Results

### The variance in rice grain yield

**Figure 1:**
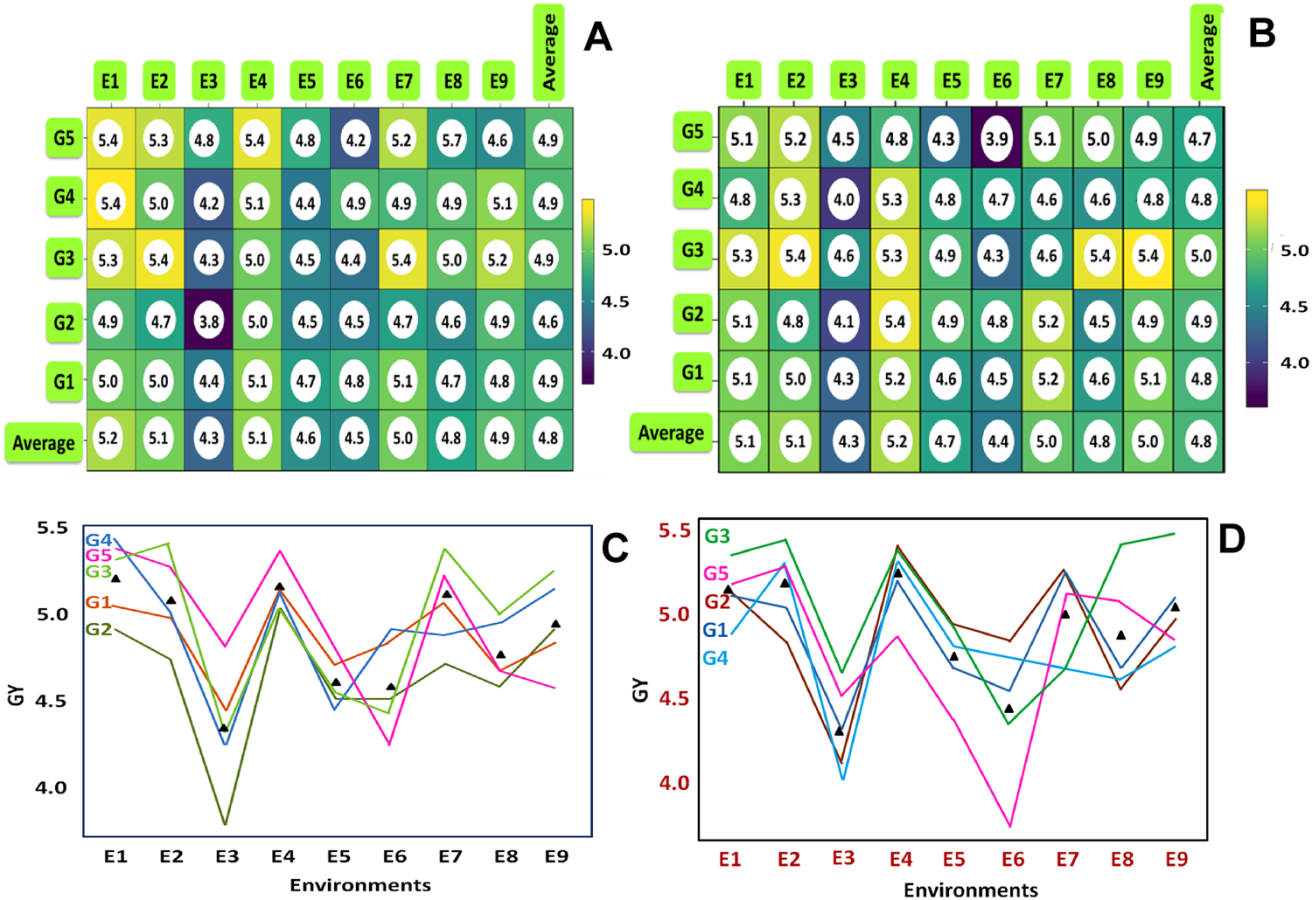
The rice grain yield (t ha^-1^) performance variation of studied five (5) rice genotypes across nine (9) environments in both AWD = Alternate wet and dry (**A**, **C**) and CF = continuous flooding (**B**, **D**) conditions.

This finding is supported by the range in the mean yield of the genotypes that were evaluated, which varied for AWD from 3.8 (t ha^-1^) (G2 in environment E3) to 5.4 (t ha^-1^) (G3 in environments E2 and E7, G4 & G5 in environments E1) (Fig. 1A,C). The G2 genotype was discovered to have the lowest yield at one place, E3, whereas the G4 genotype was detected at locations in E7, E1, E2, and E4 (Fig. 1). In every area, genotype G3 produced the most (Fig. 1A). The observed variation in the mean yield of the examined genotypes for CF (Fig. 1B,D) ranges from 3.9 (t ha^-1^) (G5 in environment E6) to 5.4 (t ha^-1^) (G3 in environments E2, E8, and E9). In most of the sites, the G3 genotype yield in both water regime treatments is consistent (Fig. 1A–D).

### Values of the combined and *AMMI* model's analysis of variance (ANOVA) applied to yield and yield-related characteristics

The combined and AMMI model's Analysis of Variance (ANOVA) for AWD and CF systems are shown in Tables 1 and 2. The results of the combined analysis of variance showed that the genotypes, environments and the GEI were significant (Tables 1 and 2). The current study used the analysis of the variance function of the AMMI model to analyze the yield and yield performance (PH, ET, PL, TSW, DM, and GY) of five prospective rice genotypes evaluated in nine environments throughout the dry season. The ANOVA showed significant impacts from the environment, G × E interactions, and genotype for each attribute. Based on the AMMI analysis of variance, every characteristic in systems PC1 and PC2 was determined to be significant. There was variation in the genotype rankings between settings for each feature that was being studied. Given that GEI has a considerable impact on each of the studied characteristics except for ET and PL; AMMI's model was employed in conjunction with multiplicative effects analysis to identify stable genotypes. The AMMI model with two significant IPCs is the best-predicted model^43,44^. At the 1% probability level, the first three components of PH were significant and explained 99.5% and 93.5% of the changes related to GEI for the AWD and CF water regimes, respectively. The third factor was the effective tiller (ET), which accounted for 95.8% and 94.8% of the GEI impact in the AWD and CF scenarios, respectively. Significant findings were found for each explanation at the 1% probability level. A significant component, or 89.7% and 93.9% for AWD and CF, respectively, supported the PL-related variations at the 1% probability level. The two main components of the TSW explained 89.8% and 81.2% of the GEI impact at the AWD and CF levels, respectively. In terms of DM, the GEI for the AWD and CF water regimes is accounted for by the first three interaction principal components of AMMI and three PCs with highly significant differences (*p* < 0.001), respectively. In this study, PC1 (59.8% and 66.6%) and PC2 (25.7% and 25%) each made a substantial contribution to the understanding of the G × E interaction for GY. The sums of squares for PC1 and PC2 together account for 85.5% and 91.6% of the total GEI at both the AWD and CF levels.

**Table 1:** Combined ANOVA and AMMI ANOVA applied to yield and yield-related trait values of rice genotypes tested at AWD conditions within nine (9) environments.

Source	df	Plant height (PH)			Effective tiller (ET)			Panicle length (PL)		
		MS	(%) SS explained	(%) GEI Cumulative	MS	(%) SS explained	(%) GEI Cumulative	MS	(%) SS explained	(%) GEI Cumulative
Combined ANOVA										
ENV	8	43.33**			21.91**			5.14**		
REP	2	15.31*			9.41			1.97		
GEN	4	472.54**			68.06**			8.27**		
GEN:ENV	32	23.08**			11.29			2.20		
Residuals	88	11.95			10.28			2.60		
AMMI ANOVA										
ENV	8	43.35**			21.93**			5.17**		
REP(ENV)	18	15.41*			9.45			1.97		
GEN	4	472.54**			68.06**			8.27**		
GEI	32	23.08**			11.29			2.20		
PC1	11	39.68**	59.1***	59.1***	20.06**	61.1***	61.1***	4.12*	64.2***	64.2***
PC2	9	19.46*	23.7***	82.8***	10.91*	27.2***	88.2***	1.37	17.5***	81.8***
PC3	7	11.23	10.6***	93.5***	3.88	7.5***	94.8***	1.22	12.1***	93.9***
PC4	5	9.64	6.5	100	3.07	4.2	100	0.86	6.1	100
Residuals	72	15.95			12.18			3.60		
Total	166	30.96			13.36			3.07		

Source	df	1000 Grain weight (TGW)			Days to maturity (DM)			Grain yield (GY)		
		MS	(%) SS explained	(%) GEI Cumulative	MS	(%) SS explained	(%) GEI Cumulative	MS	(%) SS explained	(%) GEI Cumulative
Combined ANOVA										
ENV	8	0.99			106**			1.411**		
REP	2	4.61**			2.93**			0.1396*		
GEN	4	97.64**			238**			0.4648**		
GEN:ENV	32	6.03**			9.24**			0.15**		
Residuals	88	1.56			3.29			153.4		
AMMI ANOVA										
ENV	8	0.99			106**			1.411**		
REP(ENV)	18	4.61**			2.93**			0.1396*		
GEN	4	97.64**			238**			0.4648**		
GEI	32	6.03**			9.24**			0.15**		
PC1	11	13.04**	74.2***	74.2***	10.8**	40.2***	40.2***	0.25**	59.8	59.8
PC2	9	3.34*	15.6***	89.8***	11.8**	36.1***	76.2***	0.13*	25.7	85.5
PC3	7	1.88	6.8	96.6	7.4**	17.5***	93.8***	105.1	9.5	95.1
PC4	5	1.30	3.4	100	3.69**	6.2	100	76.1	4.9	100
Residuals	72	2.26			4.29			173.4		
Total	166	6.21			14.4			319.5		

GEN = Genotype; ENV = Environment; MS = Mean Square; AMMI = Additive main effects and multiplicative interaction; GEI = Genotype by environment interaction; PC = Principal component; *** = Significant at 0.1%;** = Significant at 1%; ; ** = Significant at 1%; * = Significant at 5% probability level; df = Degrees of freedom.

**Table 2:** Combined ANOVA and AMMI ANOVA applied to yield and yield-related trait values of rice genotypes tested at continuous flooding (CF) within nine (9) environments.

Source	df	Plant height (PH)			Effective tiller (ET)			Panicle length (PL)		
		MS	(%) SS explained	(%) GEI Cumulative	MS	(%) SS explained	(%) GEI Cumulative	MS	(%) SS explained	(%) GEI Cumulative
Combined ANOVA										
ENV	8	142.31**			22.24**			2.7*		
REP	2	16.86**			4.71			2.02		
GEN	4	333.95**			36.81**			11.36**		
GEN:ENV	32	19.80**			8.67*			2.51*		
Residuals	88	6.54			5.43			1.56		
AMMI ANOVA										
ENV	8	142.31**			22.24**			2.7*		
REP(ENV)	18	16.86**			4.71			2.02		
GEN	4	333.95**			36.81**			11.36**		
GEI	32	19.80**			8.67*			2.51*		
PC1	11	31.15**	54.1	54.1	15.32**	60.8	60.8	3.4*	46.6	46.6
PC2	9	21.49**	30.5	84.6	7.54*	24.5	85.2	2.3*	25.9	72.4
PC3	7	13.52*	14.9	99.5	4.2	10.6	95.8	1.98	17.3	89.7
PC4	5	0.59	0.5	100	2.31	4.2	100	1.65	10.3	100
Residuals	72	8.64			6.23			2.42		
Total	166	28.11			8.51			2.64		

Source	df	1000 Grain weight (TGW)			Days to maturity (DM)			Grain yield (GY)		
		MS	(%) SS explained	(%) GEI Cumulative	MS	(%) SS explained	(%) GEI Cumulative	MS	(%) SS explained	(%) GEI Cumulative
Combined ANOVA										
ENV	8	2.53*			107.31**			1.59**		
REP	2	2.39			0.06*			0.20**		
GEN	4	128.47**			230.93**			0.36**		
GEN:ENV	32	2.88*			9.98**			0.23**		
Residuals	88	2.51			0.03			0.04		
AMMI ANOVA										
ENV	8	2.53*			107.31**			1.59**		
REP(ENV)	18	2.39			0.06*			0.20**		
GEN	4	128.47**			230.93**			0.36**		
GEI	32	2.88*			9.98**			0.23**		
PC1	11	4.49*	53.6	53.6	12.93**	44.5***	44.5***	0.45**	66.6	66.6
PC2	9	2.82*	27.6	81.2	11.84**	33.4***	77.9***	0.20**	25	91.6
PC3	7	1.91	14.5	95.7	7.40**	16.2***	94.2***	0.07	7.1	98.7
PC4	5	0.79	4.3	100	3.73**	5.8	100	0.01	1.3	100
Residuals	72	2.91			0.06			0.08		
Total	166	5.85			14.62			0.23		

GEN = Genotype; ENV = Environment; MS = Mean Square; AMMI = Additive main effects and multiplicative interaction; GEI = Genotype by environment interaction; PC = Principal component; *** = Significant at 0.1%;** = Significant at 1%; * = Significant at 5% probability level; df = Degrees of freedom.

### Genotypes and water regimes are graphically shown in the *AMMI* Biplots

**Figure 2:**
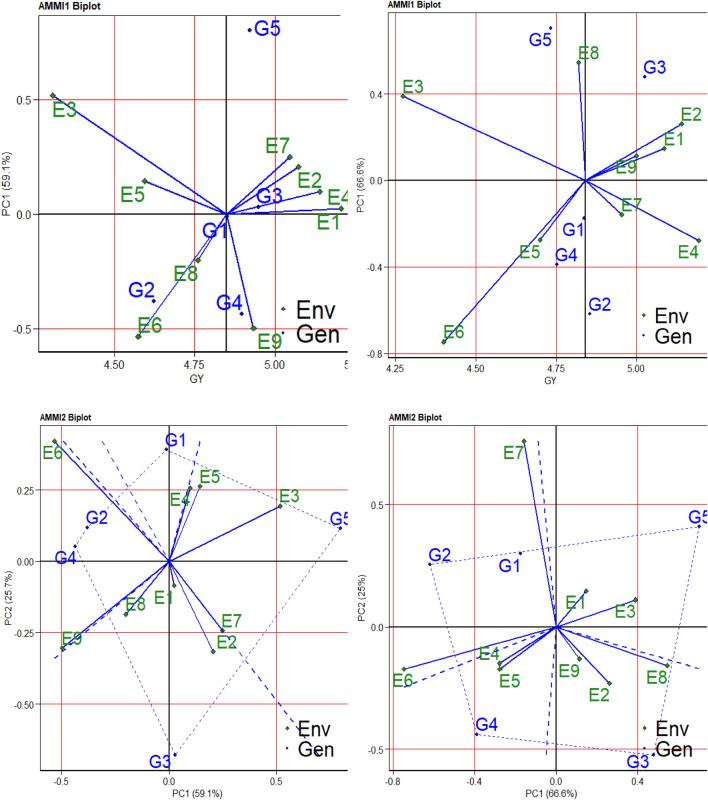
AMMI1 and AMMI2 biplot for rice grain yield (t ha^-1^) of five (5) genotypes under nine (9) diverse environments using GE IPAC scores in both AWD (**A**, **C**) and CF (**B**, **D**) conditions. G = genotype; E = environment; AMMI = Additive main effects and multiplicative interaction; PC = Principal component; AWD = Alternate wet and dry; CF = Continuous flooding.

According to the research, the distribution of genotypes and environments is represented by the AMMI1 biplot stability of yield, where PC1 accounts for 66.6% in CF systems and 59.1% in the AWD approach (Fig. 2A,B). To determine the mega-environment and stable genotypes throughout AWD and CF, biplots for AMMI1-Means vs. PC1 and AMMI2-PC1 vs. PC2 were created (Figs. 2A,B). The means of the environments and genotypes were plotted against their IPCA1 using the AMMI1 biplot. The low-yielding nature of environments E6, E3, and E5 was later shown (Fig. 2A AMMI1 Biplot). The genotypic and environmental scores that correspond to the GY IPCA1 and IPCA2 are shown in the AMMI2 biplot (Fig. 2C,D AMMI2 Biplot). The AMMI 1 biplot is typically interpreted as follows: displacements along the ordinate indicate differences in interaction effects, while displacements along the abscissa reflect variations in main (additive) effects. Zero PC1 genotypes are more adaptable to all environments and are less influenced by external variables. As evidenced by their nearly low PC1 scores, varieties G5 were the most stable and adaptive genotypes under these circumstances (Fig. 2A). However, since genotype G3's mean yield was higher than the other genotypes', it is recommended. In conclusion, the best yield might not come from a sturdy and flexible variety. The first two principal components, PC1 and PC2, could account for 84.8% of the total variance in the GEI (Fig. 2C). Regarding the grain yield attribute, there was the least amount of interaction between genotypes and environments in G1 > and G2. The AMMI1 biplot (Fig. 2B) showed that grain yields were below average for G3, G2, and G5, but they were above average for G1 and G5. G5 was the genotype that produced the least from these. Strong G × E interaction effects were seen in G5 and G3 across the genotypes under evaluation. Conversely, G1 displayed a reduced G × E interaction. The grain yield display interaction of PC1 and PC2 of the genotypes that were assessed in the eight settings is represented by the AMMI2 biplot in Fig. 2D. Yan and Kang^45^ suggest that distances from the biplot origin are a useful measure of the degree of genotype or environment interaction. While they did not exert considerable interaction forces, the environmental vectors with short spokes in E1, E9, E3, E4, and E5 in this figure greatly contributed to the genotype's stability. The environmental vectors with long spokes, on the other hand, show high interaction. As a result, environments E7 and E8 had long spokes, demonstrating their high discriminating ability.

### The examination of rice grain yield stability

Every genotype in the CF and AWD systems performed very identically in each of the nine settings (Tables 3 and 4). Table 3 shows that, in all circumstances, G3 and G2 had the highest and lowest grain yields, respectively. The greatest and lowest mean values (4.95 to 4.62 t ha^-1^) of the variation in rice grain yields across the five rice genotypes were similar to one another. Table 4 displays the genotype means, which varied between 5.03 and 4.73 t ha^-1^ depending on the environment. Tables 3 and 4 show that all genotypes performed better in AWD than in the CF system.

**Table 3:** The grain yield (t ha^-1^) of five tested rice genotypes at AWD conditions in nine different environments.

Environments	Genotypes					Genotypes mean
	G1	G2	G3	G4	G5	
E1	5.03	4.90	5.30	5.43	5.37	5.21
E2	4.97	4.73	5.40	5.00	5.27	5.07
E3	4.43	3.77	4.30	4.23	4.80	4.31
E4	5.13	5.03	5.03	5.13	5.37	5.14
E5	4.70	4.50	4.53	4.43	4.80	4.59
E6	4.83	4.50	4.40	4.90	4.23	4.57
E7	5.07	4.70	5.37	4.87	5.23	5.05
E8	4.67	4.57	4.97	4.93	4.67	4.76
E9	4.83	4.90	5.23	5.13	4.57	4.93
Genotypes mean	4.85	4.62	4.95	4.90	4.92	4.85

**Table 4:** The grain yield (t ha^-1^) of five tested rice genotypes at continuous flooding (CF) in nine different environments.

Environments	Genotypes					Genotypes mean
	G1	G2	G3	G4	G5	
E1	5.07	5.13	5.10	5.30	4.83	5.09
E2	5.00	5.23	4.80	5.40	5.27	5.14
E3	4.27	4.47	4.07	4.60	3.97	4.27
E4	5.17	4.83	5.37	5.33	5.27	5.19
E5	4.63	4.33	4.90	4.87	4.77	4.70
E6	4.50	3.70	4.80	4.30	4.70	4.40
E7	5.20	5.07	5.23	4.63	4.63	4.95
E8	4.63	5.03	4.50	5.37	4.57	4.82
E9	5.07	4.80	4.93	5.43	4.77	5.00
Genotypes mean	4.84	4.73	4.86	5.03	4.75	4.84

**Figure 3:**
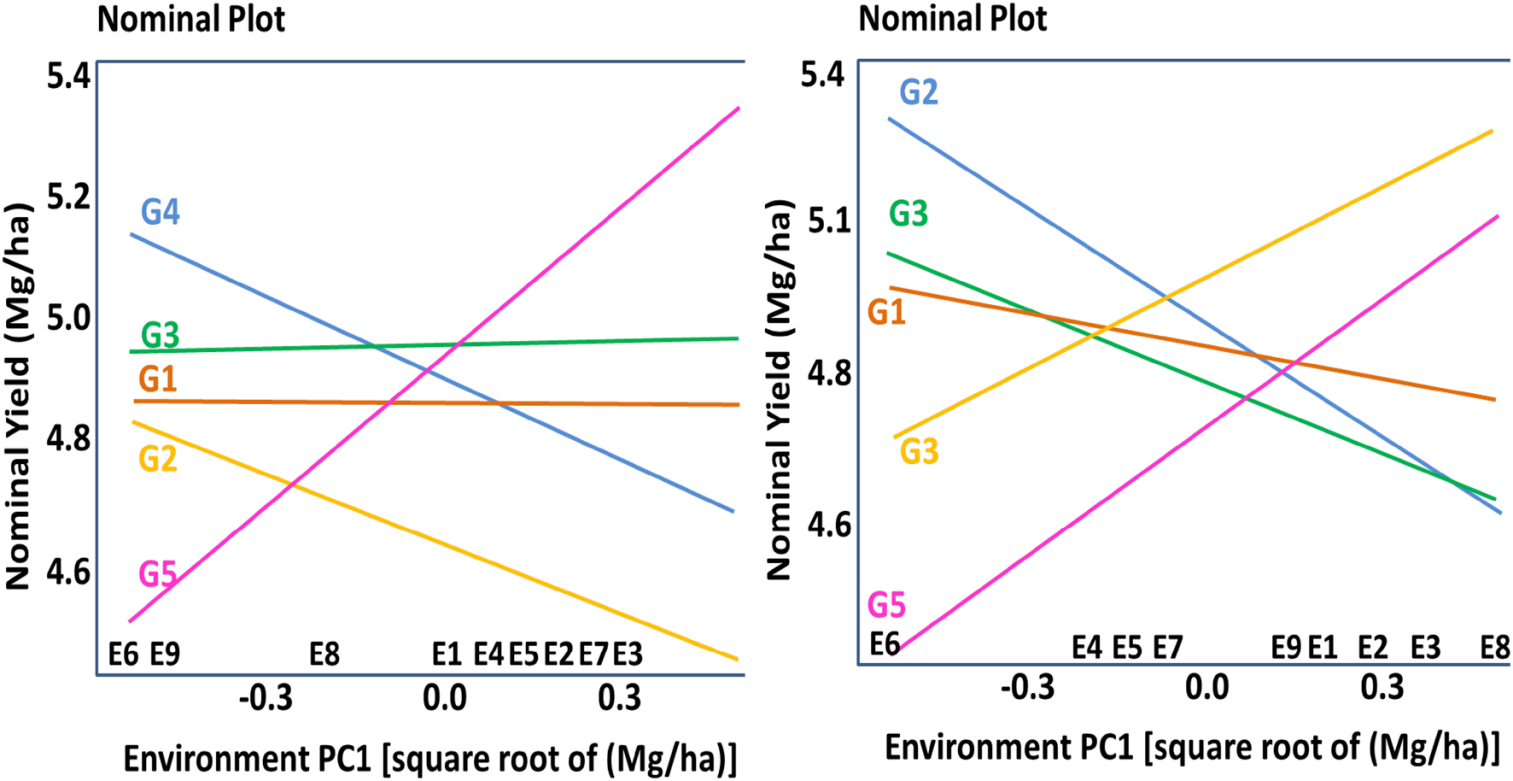
AMMI Biplot nominal plot of five (5) rice genotypes with yield and stability in nine (9) different environments in both AWD (**A**) and CF (**B**) conditions. G = genotype; E = environment; IPCA 1 = Interaction principal component axis 1; G1 = Binadhan-10, G2 = Binadhan-8, G3 = BRRI dhan29, G4 = BRRI dhan47, G5 = Bina dhan-17.

Using the AMMI biplot adaptation map (Nominal plot) for genotypes or generic genotypic adaptation. According to the AMMI biplot adaption map of yield, the AWD approach was more suited for G1 (Binadhan-10) and G3 (BRRI dhan-29). These two genotypes exhibited comparable performance in all situations. Although it varies from environment to environment, G5 (Binadhan-17) performed best in environment-3 (Union: Kolma). Only the G1 (Binadhan-10) exhibited reduced G × E interaction in the CF system. Similar to the AWD approach, Binadhan-17 also had the largest G × E interaction in this instance (Fig. 3).

### Using the which-won-where method of the polygon of GGE biplot methodology, the specific genotypic adaption

**Figure 4:**
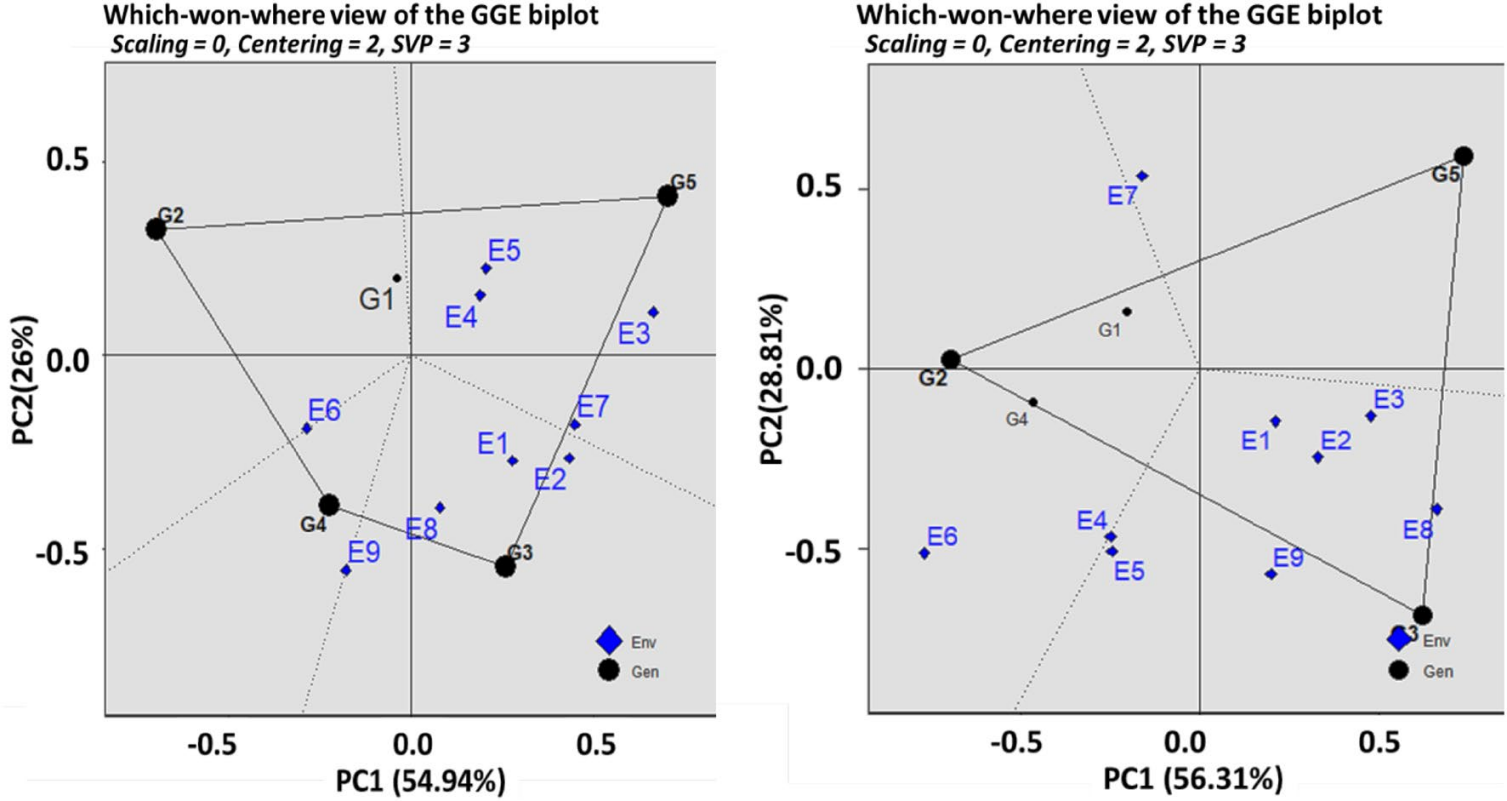
Polygon of GGE Biplot for clustering environments in both AWD (**A**) and CF (**B**) conditions. G = genotype; E = environment; PC = Principal component). G1 = Binadhan-10, G2 = Binadhan-8, G3 = BRRI dhan29, G4 = BRRI dhan47, G5 = Bina dhan-17.

The vertex genotype in the AWD approach was G4 (BRRI dhan47), while the genotype with the highest responsiveness was G2 (Binadhan-8), per the study's Polygon of the GGE biplot of rice grain yield. In the CF system, G5 (Binadhan-17) has been found in both responsive and vertex genotypes. The genotype main effects and the GE interaction, which were 54.94% and 56.31%, respectively, were explained by the PC1 in the AWD technique and in the CF system (Fig. 4).

### GGE biplot genotype view with the average environment coordination (AEC) of rice grain yield

**Figure 5:**
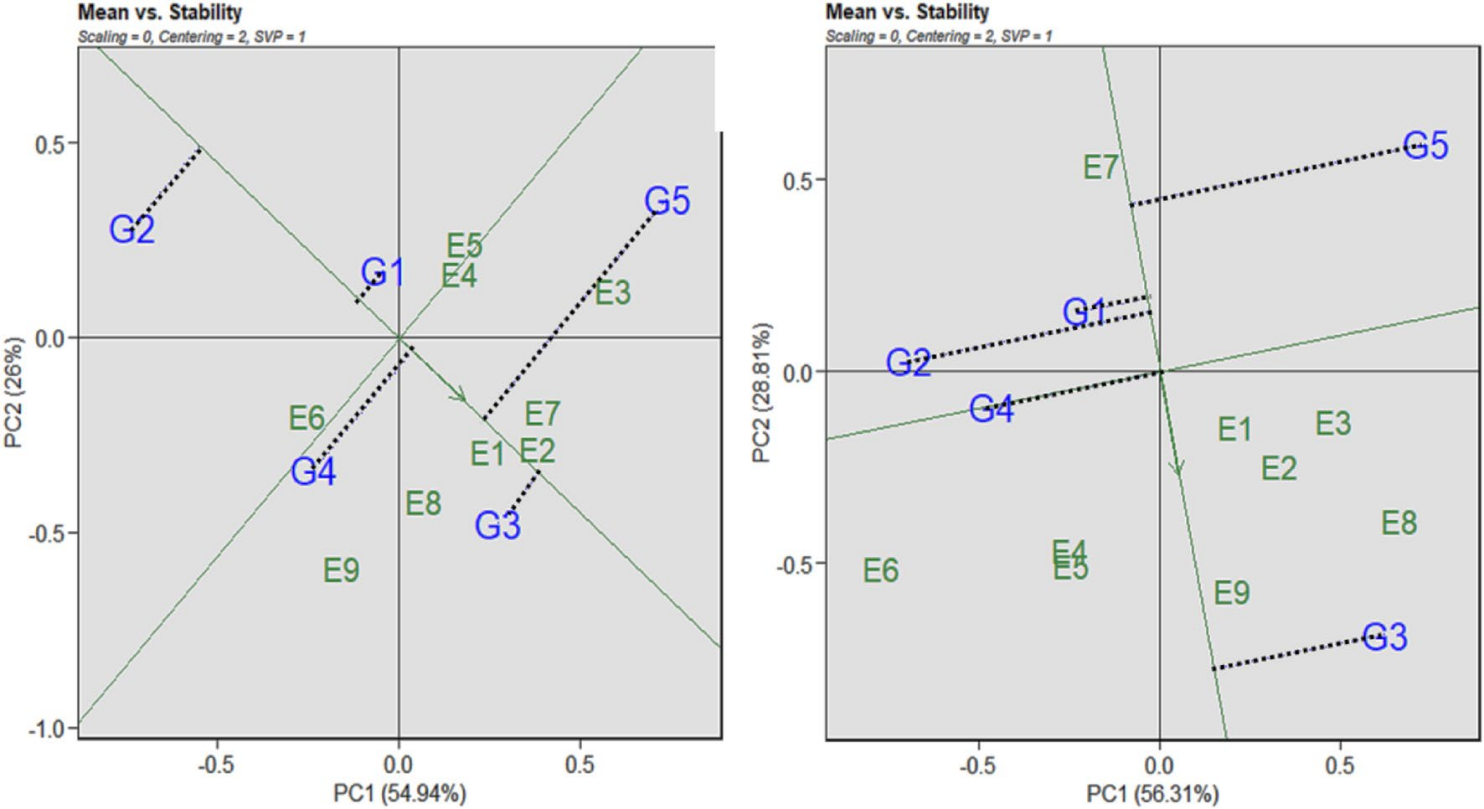
Assessment of five (5) inbred lines of rice according to their yield and stability in nine different environments in both AWD (**A**) and CF (**B**) conditions. G = genotype; E = environment; GGE = Genotype by environment biplot; PC = Principal component; G1 = Binadhan-10, G2 = Binadhan-8, G3 = BRRI dhan29, G4 = BRRI dhan47, G5 = Bina dhan-17.

Figure 5A,B show the genotype ranking according to mean performance and stability. According to previous research, PC2 (a measure of instability) of a GGE biplot must approximate the GE effects associated with each genotype if PC1 of the biplot approximates the genotype's main effects^34,46^. The average environment coordinate (AEC) axis, which is defined by the average PC1 and PC2 scores of all the environments, is the line that goes through the biplot origin and the average environment. The yield stability of genotypes was assessed using the AEC approach, which uses the average principal components in all conditions. Genotype G3 (BRRI dhan29) exhibited the highest grain yield and excellent stability under Alternate Wetting and Drying (AWD) conditions, as depicted in Fig. 5A. Conversely, genotype G1 (Binadhan-10) demonstrated average grain yield but maximum stability. Genotypes G4 (BRRI dhan47) and G5 (Binadhan-17) showed grain yields close to that of G3 (BRRI dhan29) but with lower stability.

Overall, it is inferred that genotypes G1 (Binadhan-10) and G3 (BRRI dhan29), offering comparable grain yields to optimal genotypes while maintaining sufficient stability, are the most desirable. Concerning the continuous flooding system (CF), genotype G3 (BRRI dhan29) displayed a nearly average grain yield, while G4 (BRRI dhan47) exhibited a high grain yield with lower stability. In contrast, G1 (Binadhan-10) showed average grain yield with the highest stability. Genotypes G2 (Binadhan-8) and G5 (Binadhan-17) were associated with the lowest grain yield and stability. In conclusion, Binadhan-10 and BRRI dhan29 emerge as the superior genotypes, demonstrating grain yield comparable to the optimal genotype while exhibiting adequate stability across conditions.

### Analyzing genotypes in relation to the ideal genotype

**Figure 6:**
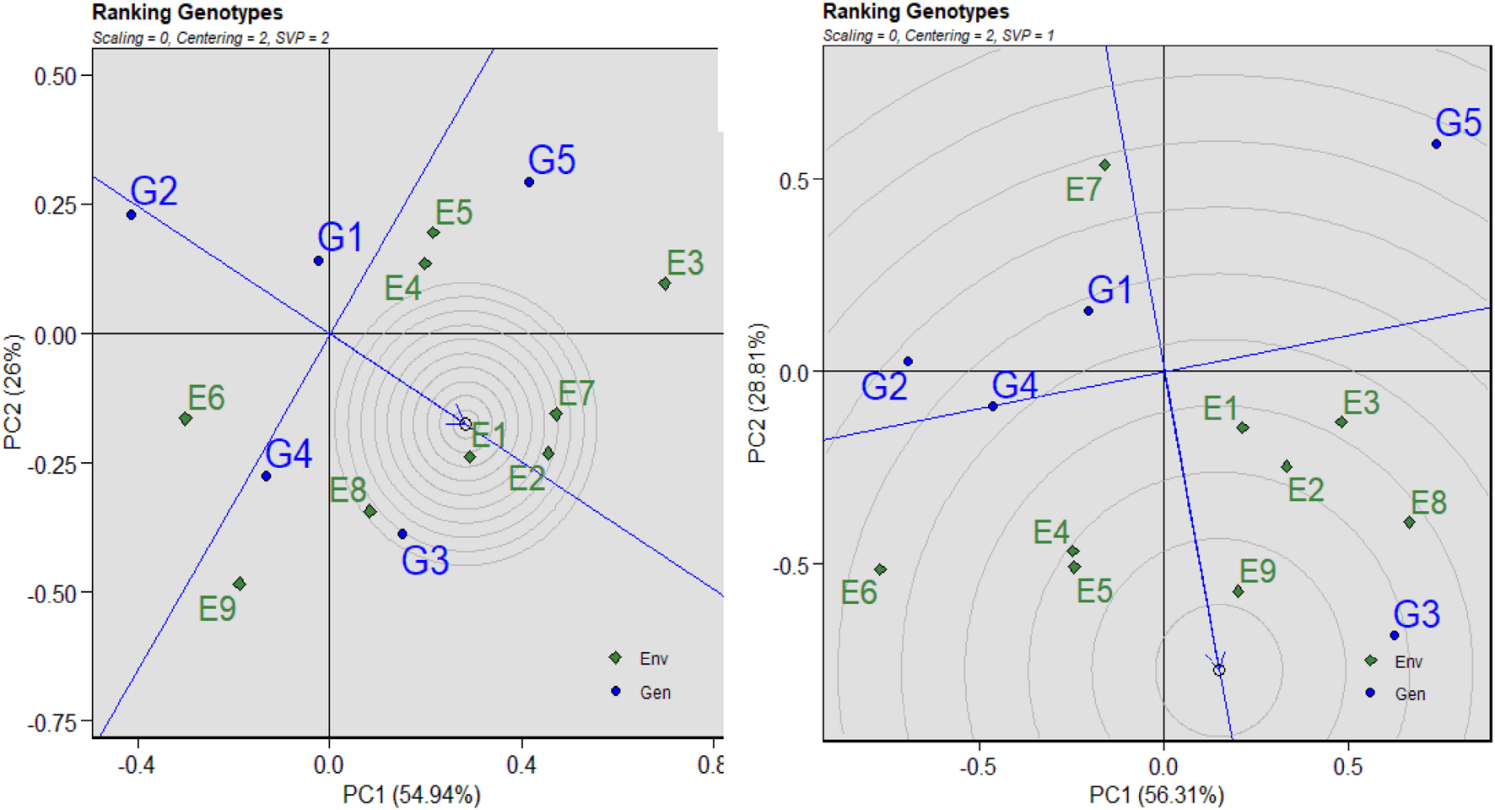
GGE biplot graph based on genotype-focused scaling for comparison of genotypes with an ideal genotype in both AWD (**A**) and CF (**B**) conditions. G = genotype; E = environment; PC1 = Interaction principal component axis 1; G1 = Binadhan-10, G2 = Binadhan-8, G3 = BRRI dhan29, G4 = BRRI dhan47, G5 = Bina dhan-17.

The GGE biplot model is an interesting tool for evaluating genotypes against an ideal genotype. Concentric circles were added to a genotype-focused scaling GGE biplot graph^35,47^ to more clearly illustrate the difference between genotypes and the ideal genotype. The GGE biplot graph (Fig. 6A,B) demonstrates that genotype G3 was positioned first in the concentric circle in both the AWD and CF techniques. Therefore, G3 was the best genotype position—better than any other rice genotype—and was followed by G4, G1, and G3. The genotype-environment interaction shown in Fig. 6A and the GGE biplot analysis of rice grain yield indicate that G3 (BRRI dhan-29), which is nearly the ideal genotype theoretically, is the best genotype. They were therefore regarded as being outside of Fig. 6B's ideal inbred group. G5 (Binadhan-17) in the CF system and the inbred G2 (Binadhan-8) in the AWD technique, on the other hand, were found to be extremely distant from the optimal genotype.

### Assessment of environment in comparison to ideal environment

**Figure 7:**
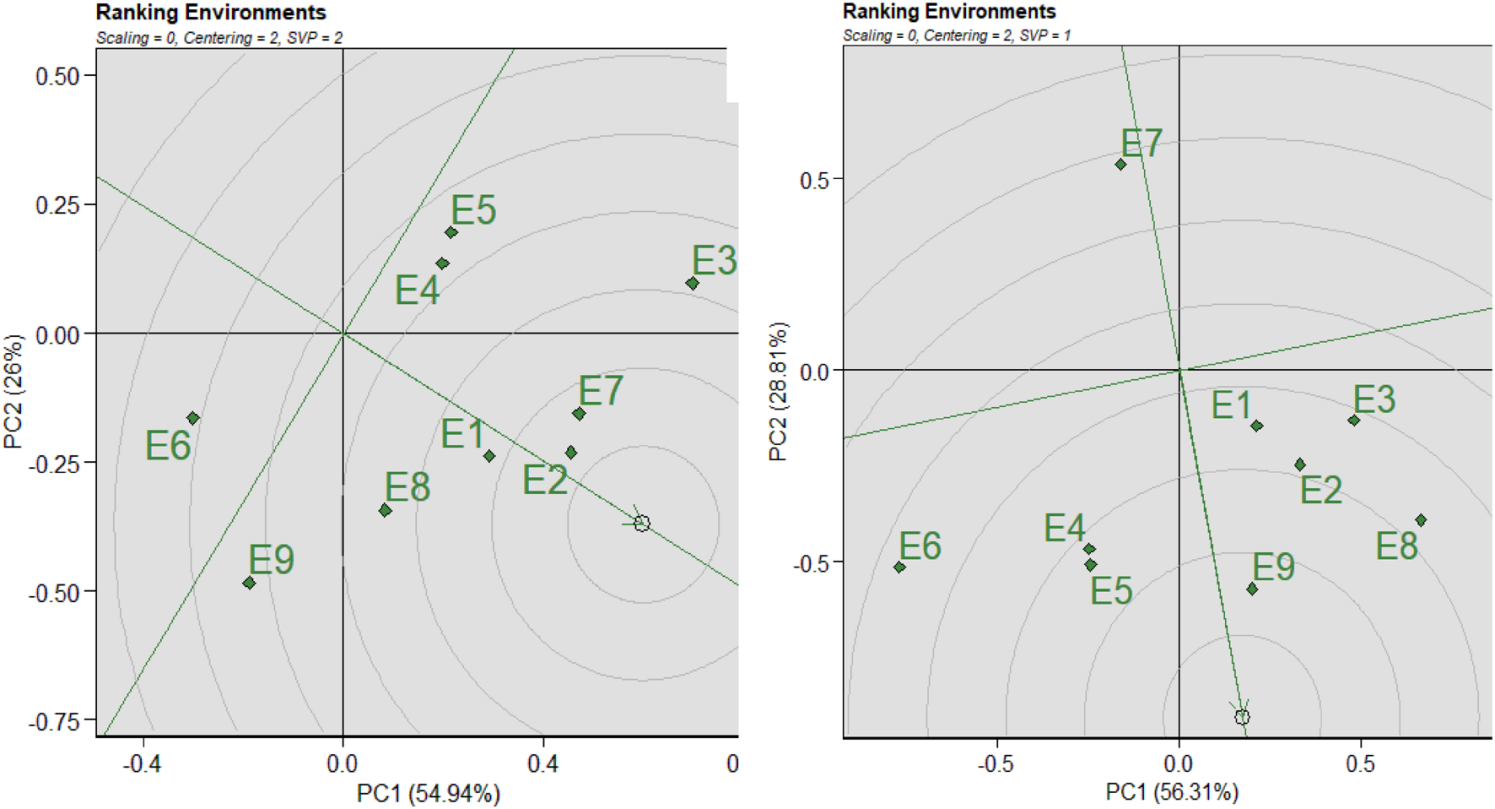
GGE biplot graph based on environment-focused scaling for comparison of environments with an ideal environment in both AWD (**A**) and CF (**B**) conditions. G = genotype; E = environment; PC1 = Interaction principal component axis 1; G1 = Binadhan-10, G2 = Binadhan-8, G3 = BRRI dhan29, G4 = BRRI dhan47, G5 = Bina dhan-17.

In optimal conditions, the longest vector—which had a low IPCA—was discovered to lie inside the centers of concentric circles. The environment-focused GGE biplot's second concentric circle depicts the ideal environment, and the settings that are most like it are the ones that are desired. In this sense, E2 is in the second concentric circle and has been in the ideal environment (Fig. 7A). Though they may be viewed as unfavorable (having fewer examples) for selecting cultivars that are widely adapted, the E6 and E9 environments were far from ideal conditions, thus selecting particularly suitable cultivars may benefit from them. Despite not being statistically representative, the most discriminating samples in the current analysis were E1, E2, E6, and E7 from Fig. 7A. Their long projection against the ATC Y-axis and distance from the plot source for the AWD approach were probably the causes of this. Consequently, E9 and E5, which represent the ideal conditions based on this research, are shown in Fig. 7B. An environment that is most representative of all environments and most effective at differentiating between genotypes is considered optimal. Similarly, because they were closer to the ideal environment, E4, E2, and E8 were judged to be the second strongest in terms of genotype discrimination (Fig. 7B). On the other hand, environment E7 was found to be distant from the ideal environment and was believed to be less successful in distinguishing between genotypes (Fig. 7B). On the E9, the standard environment is displayed. Figure 7B demonstrated that while E5, E6, E8, and E9 were not statistically representative in the current investigation, they were the most discriminating because they showed the longest projection over the ATC Y-axis and were located farthest from the plot source for the CF technique.

### Correlation between various stability statistics

**Figure 8:**
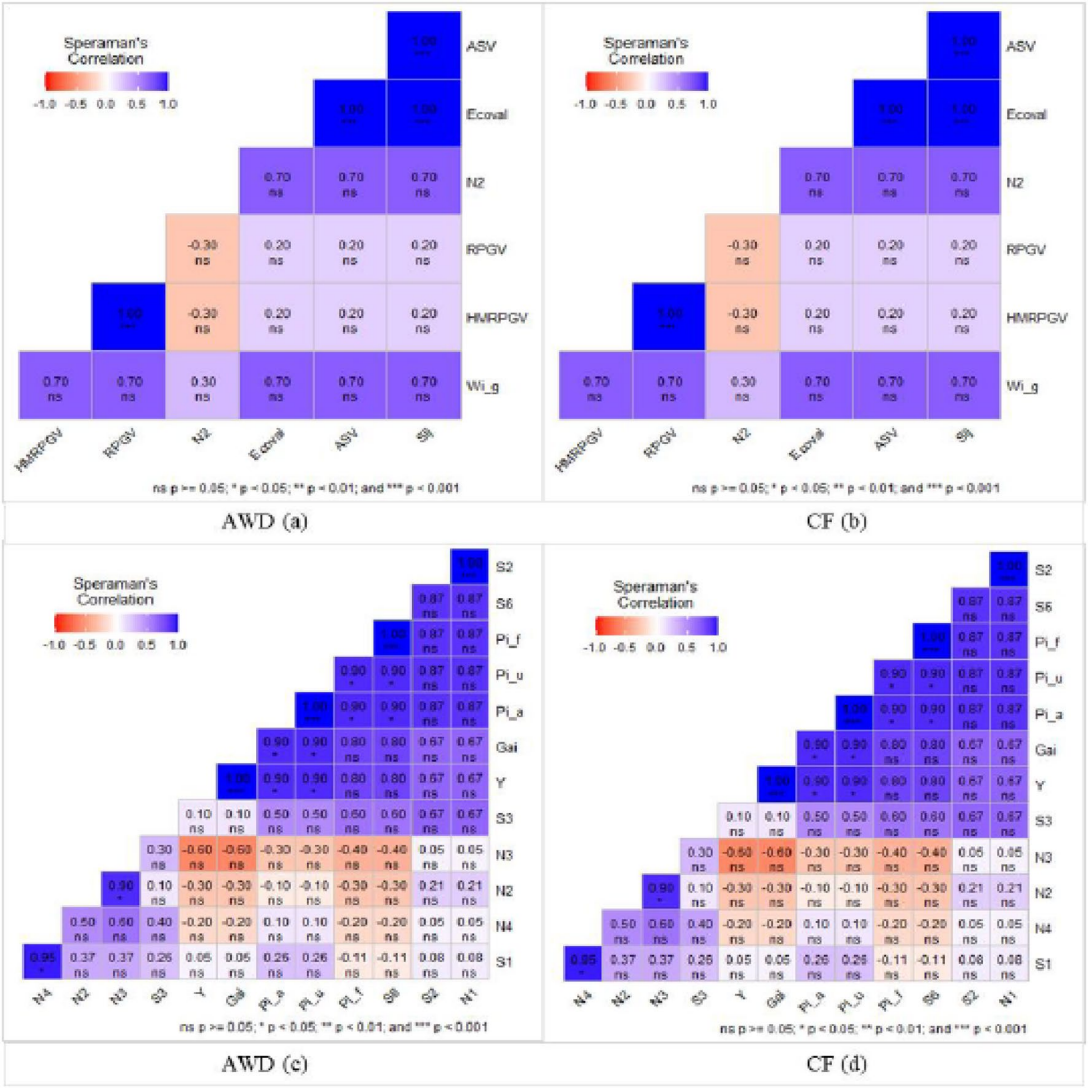
Heatmap-based Speraman’s correlation coefficient among various stability parameters with yield data of 5 rice genotypes evaluated at 9 test environments. Parametric measures such as: ASV–AMMI stability value, RPGV: Relative performance of genotypes values; HMRPGV: Harmonic mean of RPGV, Ecoval: Wricke's ecovalence. Non-parametric measures such as: N1, N2, N3, N4: Thennarasu"s statistics, Sij: Deviations from the joint-regression analysis, S1: mean of the absolute rank differences of a genotype over the n environments, S2: variance among the ranks over the k environments, S3: sum of the absolute deviations, S6: relative sum of squares of rank for each genotype, Gai: Geometric adaptability index, Pi_a, Pi_f, Pi_u: Superiority indexes for all, favorable and unfavorable environments, respectively.

To determine the relationship between each pair of AMMI stability parameters, Spearman's correlations (Fig. 8) discovered a strong association between ASV and sij, Ecoval and sij, ASV and Ecoval, HMRPGV, and RPGV. In both AWD and CF systems, ASV, sij, HMRPGV, and RPGV exhibited a strong correlation with each other, while the other measures showed a non-significant correlation. The results showed that in both the CF and AWD systems, the same parameters showed a significant correlation in the same patterns. A heatmap-based Speraman's correlation coefficient was used to analyze the relationships between mean grain yield and the calculated stability indicators (Fig. 8). The results demonstrated a significant and positive association between the mean yield and S1, S2, S6, N1, N3, Gai, and Y as well as Pi_f, Pi_u, and Pi_a. It was demonstrated that there was a positive and significant correlation between the stability statistics N1 with S2, S6 with Pi_f, S6 with Pi_u, S6 with Pi_a, Pi_f with Pi_u, Pi_f with Pi_a, Pi_u with Pi_a, Pi_u with Gai, Pi_u with Y, Pi_a with Gai, and N4 with S1.

### The multi-trait genotype-ideotype distance index (MGIDI) selection process and selection gains

**Figure 9:**
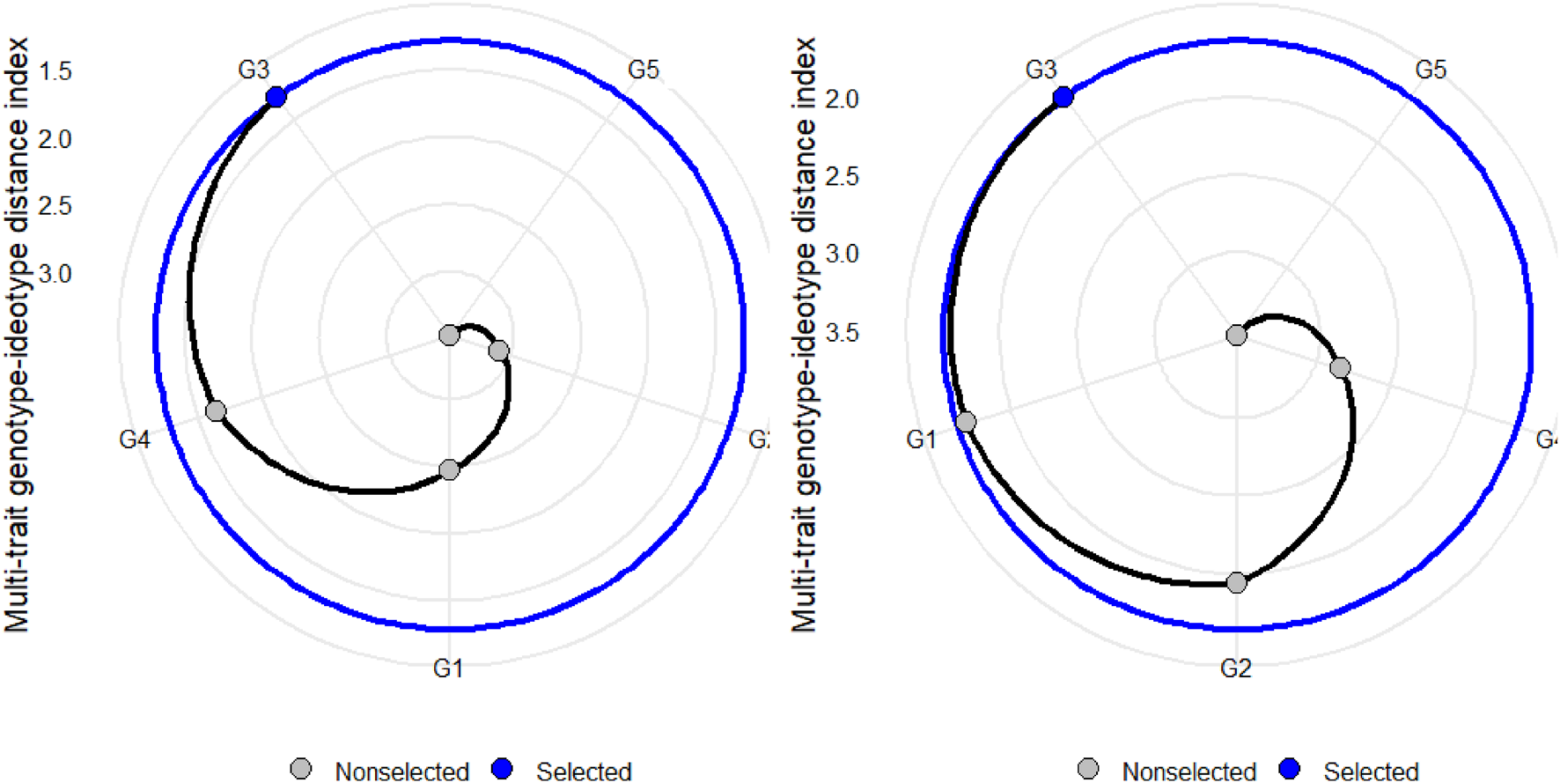
A Genotype ranking in ascending order for the MGIDI index in both AWD (**A**) and CF (**B**) conditions. The selected genotypes based on this index are shown in blue. The central blue circle represents the cut point according to the selection pressure. G = genotype; G1 = Binadhan-10, G2 = Binadhan-8, G3 = BRRI dhan29, G4 = BRRI dhan47, G5 = Bina dhan-17.

The MGIDI function makes it easier to compute the index, allowing for identifying idiotypes by declaring which traits were desired to increase or decrease^48^. The lower contribution of a factor in the genotype indicated that the genotype is highly selective for the trait in a particular factor. The factors closer to the edge in Fig. 9 are less distant from the desired idiotype, and this can be used for genotype selection.

To identify the genotypes that conserve water, the multi-trait genotype-ideotype distance (MGIDI) index was calculated, accounting for all evaluated features. A significant genotypic influence was found for each of the five features that were measured: G1 (Binadhan-10), G2 (Binadhan-8), G3 (BRRI dhan-29), G4 (BRRI dhan-47), and G5 (Binadhan-17) (Fig. 9A,B). The analytical results showed that G3 (BRRI dhan-29) was selected for both AWD & CF based on traits, such as plant height, effective tiller, panicle length, 1000 grain weight, life duration, plot yield, and grain yield.

The analysis involving two main factors from a set of six traits delineates their collective contribution to 58.4 percent of the total variance among traits, revealing specific trait clusters. These factors offer a simplified view of trait interconnections for AWD: Factor 1 includes traits such as ET, DM, and GY, while Factor 2 consists of PH, PL, and TGW for AWD. Communal and unique attributes account for 86.6% and 32.4%, respectively, of all the genetic diversity within the dataset, according to Table 5. This supports the effectiveness of factor analysis in creating an index that facilitates optimal trait selection^41^. The use of the MGIDI index resulted in favorable genetic enhancements across all examined traits, with an overall genetic gain of 13.06 percent. Notable traits like TGW and ET showed impressive selection gains of 4.78 and 5.55 percent, respectively (Table 6). This highlights the potency of the MGIDI index in promoting precise and advantageous trait selection, thereby improving crop development strategies.

**Table 5:** Eigenvalues, explained variance, factorial loadings after varimax rotation, and communalities obtained in the factor analysis within the soil moisture regime based on MGIDI values for AWD.

VAR	FA1	FA2	Commu nality	Unique nesses	Xo	Xs	SD	SDperc	h2	SG	SG (%)	sense	goal
ET	− 0.89	0.2	0.83	0.17	12.8	14.9	2.12	16.6	33.5	0.711	5.55	Increase	100
DM	− 0.96	0.04	0.93	0.07	144	149	4.62	3.2	74	3.42	2.37	Increase	100
GY	− 0.8	0.07	0.64	1.41	4.85	4.91	0.057	1.18	12.9	0.00734	0.151	Increase	100
PH	0.26	− 0.89	0.87	0.13	97.3	97.5	0.194	0.199	72	0.14	0.143	Increase	100
PL	− 0.12	− 0.94	0.9	0.1	24.9	25	0.114	0.458	15.2	0.0173	0.0697	Increase	100
TGW	0.66	− 0.74	0.99	0.01	24.9	23.3	− 1.58	− 6.34	75.4	− 1.19	− 4.78	Increase	0
Average			0.86	0.32									
T. decrease											− 4.78		
T. Increase											8.237		
Eigenvalues	3.5	1.65											
Variance (%)	58.4	27.4											
Cumulative variance (%)	58.4	85.9

**Table 6:** Eigenvalues, explained variance, factorial loadings after varimax rotation, and communalities obtained in the factor analysis within the soil moisture regime based on MGIDI values for CF.

VAR	FA1	FA2	Commu nality	Unique nesses	Xo	Xs	SD	SD (%)	h2	SG	SG (%)	Sense	Goal
ET	− 0.93	0.25	0.93	0.07	12.3	13.7	1.41	11.5	29.8	0.419	3.42	Increase	100
DM	− 0.96	0.27	0.98	0.02	24.1	25	0.874	3.62	28.9	0.253	1.05	Increase	100
GY	− 0.99	0.03	0.98	0.02	4.84	4.91	0.0728	1.5	6.71	0.00488	0.101	Increase	100
PH	− 0.09	− 1	1	0	96.1	94	− 2.07	− 2.15	62.7	− 1.3	− 1.35	Increase	0
PL	0.41	− 0.87	0.93	0.07	24.7	22.2	− 2.53	− 10.2	83.2	-2.11	− 8.52	Increase	0
TGW	− 0.57	0.75	0.89	0.11	144	149	4.52	3.14	72.7	3.29	2.28	Increase	100
Average			0.95	0.05									
T. decrease											9.87		
T. Increase											6.851		
Eigenvalues	4.13	1.58											
Variance (%)	68.8	26.4											
Cumulative variance (%)	68.8	95.1											

**Figure 10:**
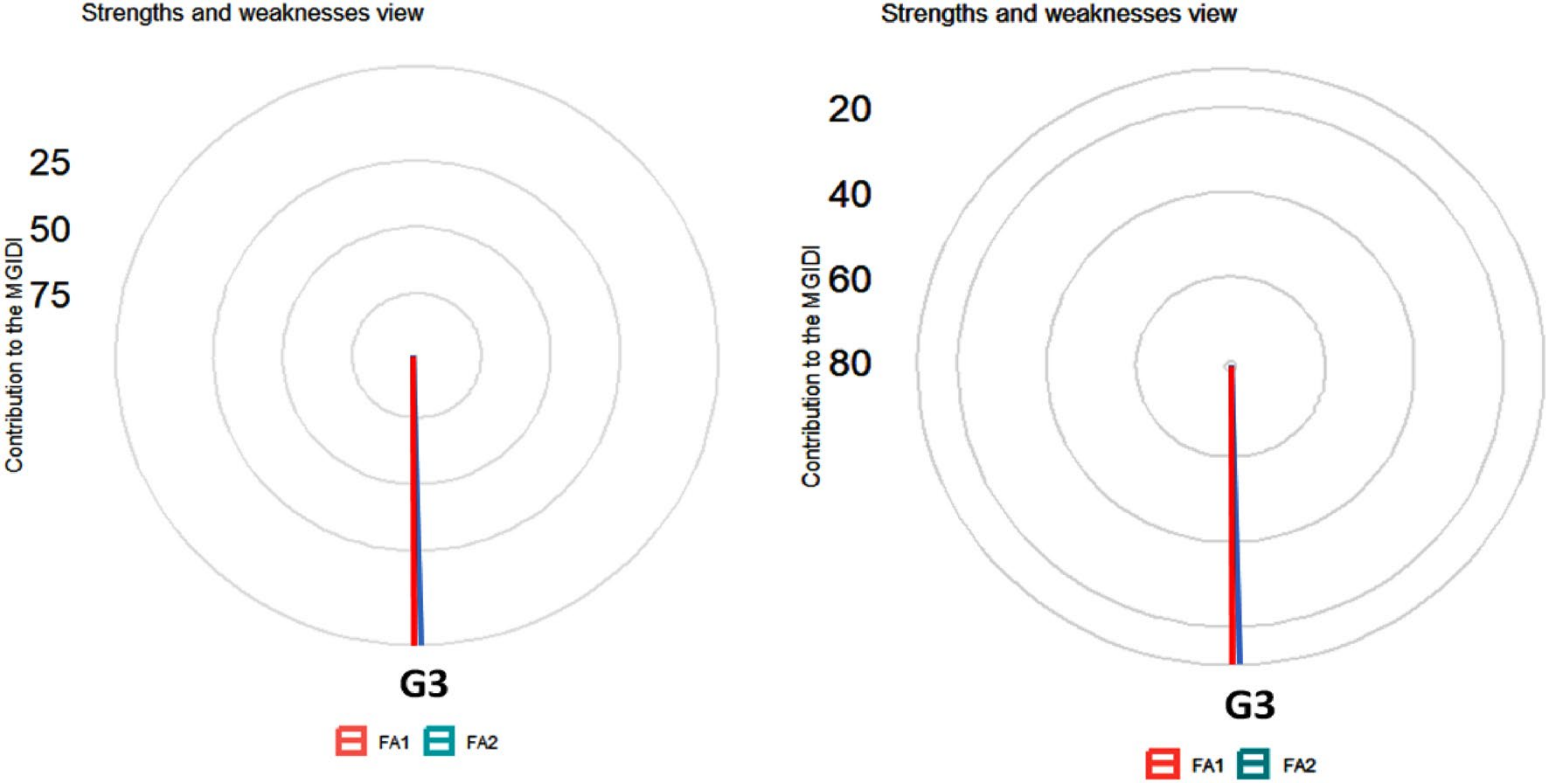
The strengths and weaknesses view of the selected rice genotype is shown as the proportion of each factor on the computed MGIDI values over two moisture regimes, namely, (**A**) AWD and (**B**) CF.

The radar plot visualizations (Fig. 10A,B) provide a detailed analysis of the strengths and weaknesses of various rice genotypes across two moisture conditions. These figures are instrumental in evaluating the genotypes' adaptation by examining how each trait contributes to the Modified Genotypic Ideotype Deviation Index (MGIDI). Each factor's contribution towards the MGIDI is strategically ranked, with the most influential factors positioned closer to the center of the plot and the less influential ones farther from the center. Traits that are closer to the outer edge of the radar plot are considered more aligned with the ideotype. This implies that a smaller proportion of the genotype variation is explained by these factors, indicating a closer similarity to the ideotypical traits. A view on strengths and weaknesses under AWD revealed that the performance of the selected genotypes, viz., G3 showed strengths related to factor FA1 that holds ET, DM, and GY, while Factor 2 consists of PH, PL, and TGW with positive SGs except TGW (Fig. 10A). This analysis provides a comprehensive understanding of how each genotype performs under varying moisture regimes and which traits contribute most effectively towards achieving an ideotype-like phenotype. This systematic approach aids in identifying key areas of strength and potential improvement for rice breeding programs under different environmental conditions. Similarly, under CF conditions (Fig. 10B), most of the selected genotypes that is G3 showed strengths related to all the factors (FA1 and FA2). According to FA1, genotype G3 performed very well with a maximum contribution towards the MGIDI.

The examination of two primary factors from an initial group of six traits uncovered that they account for 68.8 percent of the variation among these traits, showcasing distinct clusters of attributes. This analysis narrows down the complexity of trait interrelations specifically for CF: Factor 1 (FA1) includes traits such as ET, DM, and GY, while Factor 2 comprises characteristics like PH, PL, and TGW in CF. The analysis displayed an average communality and uniqueness representing 95.3% and 5.6% of the genetic variance, as detailed in Table 6, affirming the utility of factor analysis in forming an index conducive to optimal trait selection^41^. Further, the implementation of the MGIDI index yielded favorable genetic improvements across the evaluated traits, achieving a total genetic gain of 16.52 percent. Particularly, traits like PL and ET marked significant selection gains of 8.52 and 3.42 percent, respectively (Table 6). This underscores the success of the MGIDI index in promoting specific and beneficial trait selection for advancing crop improvement strategies.

## Discussions

For irrigated rice, the AWD irrigation system was created as a way to save water. The method has naturally been used mostly in places with limited water resources. In this study, water treatment had a dominant effect on grain yield, in addition to the effect of the cropping season, which was significant due to climatic changes. The International Rice Research Institute (IRRI) created the AWD technique, which was first presented in Bangladesh in 2004, to meet the demands of farmers who want to reduce their use of fuel, water, and energy in their irrigated rice production. Lowland rice producers may utilize the AWD, a water-saving technique, to use less water in their irrigated crops. AWD involves flooding the land with irrigation water a certain number of days after the ponded water evaporates. Water management at saturated soil conditions was shown to sustain similar moisture content in the soil, thus supporting leaf physiological and plant growth performances that resulted in maintaining a high grain yield of rice. In AWD, the interval between irrigations might range from one day to more than ten days during which the land is not watered. "Safe AWD" refers to the water depth threshold of 15 cm (below the surface) before irrigation, as it prevents yield reduction. Water savings with Safe AWD range from 15.0 to 30.0 percent. The country's north-central, northwestern, and southwestern regions face even more pressing problems due to falling groundwater levels and growing water shortages. Thus, there is little doubt that the intense groundwater abstraction caused by dry season rice cultivation is responsible for the groundwater levels' decline, which is occurring at a rate of 0.1–0.5 m/yr^49^. The majority of morpho-physiological and agronomical features of rice cultivation are significantly impacted adversely by heat and/or drought, and this is reflected negatively in grain production, particularly if these stressors occur during crucial stages of the plant life cycle. Therefore, the creation of cultivars with high productivity together with high stability under fluctuating drought and/or heat levels is the primary focus of plant breeding specialists and the long-term goal of modern breeding programs^50,51^. Thus, in the present investigation, genotypes with high adaptability and stability are chosen using AMMI and GGE models. The environment and its interactions have a significant impact on yield and yield-related characteristics, therefore understanding the G × E interaction is crucial for identifying stable genotypes in breeding programs.

The yield component characteristics PH, ET, PL, TGW, and DM were very significant, which is consistent with the findings of Yadav et al*.*^52^. This suggests that each genotype is genetically diverse and reliable for character selection of these variables. The results of the ANOVA study showed that there were significant differences in the variances related to genotype, environment, and genotype × environment interaction for the characters PH, ET, PL, TGW, and DM. Literature has also shown corroborative results across genotypes in rice^53–55^ *Zea may* ^56^, mung bean^57^ and *A.* spp.^58–65^. The noteworthy interplay resulting from G × E serves as a gauge for the diverse reactions of rice genotypes to quantitative attributes in both scenarios. This indicates that to identify genotypes with either particular adaptation or broad adaptability, genotypes must be assessed for stability analysis^66^. The genetic variety of the genotypes, environmental factors, and the interactions between the genotypes and environments might all be attributed to the extremely significant variations in environments and genotypes found in this research. Numerous studies have shown comparable findings, and the variety of the multiple conditions examined with the various genotypes planted may account for the extremely substantial GEI for certain agronomic variables^67–69^. Numerous studies have shown that GEIs significantly affect rice yields^51,70,71^.

The AMMI model, which is the popular stability analysis method, measures the amount of variability in both genotype and environment using a combination of ANOVA and GEI^44,72^. The main effects (genotype and environment means) are shown by the X-coordinate in the AMMI 1 biplot (Fig. 2 A–D), while the interaction effects (PC1) are represented by the Y-coordinate. Significant differences exist across genotypes in the AMMI1 biplot concerning direction and amplitude along the X-axis (yield) and Y-axis (PC 1 scores). A larger score (absolute value) implies instability and is specific to particular situations when evaluating a biplot experiment. Main effects with a PC score around zero suggest low interaction effects and are stable^73^. A positive interaction occurs between a genotype and its environment when their signals on the PC axis match; a negative interaction occurs when their signs disagree^74^. Genotypes with a PC score of almost zero and a root average yield larger than the grand mean, according to the AMMI model, are often able to adapt to any environment. Nonetheless, it is believed that genotypes with high IPCA scores and mean performances are better suited to their surroundings^47^. AWD G3 showed particular flexibility for E7, E2, and E4 conditions with mean yields below the grand mean, as shown in Fig. 2A. The AMMI1 biplot explained the distribution of the tested genotypes based on the GY mean and PC1 values, allowing for the identification of the genotypes G3 and G2, which had the greatest GY mean and the lowest IPCA1 scores (Fig. 2B). The association between genotypes and environments and their environmental adaptability is shown by the PC score of genotypes^75^. The most stable genotypes are those with an IPCA score close to zero^76^. A genotype is more stable, according to Balakrishnan^77^, if its IPCA score is closer to zero. Conversely, a high IPCA score positive or negative indicates that the genotype has adapted to a certain environment. The AMMI2 biplot provided more explanation for the multiplicative effects of GEI throughout the first two IPCAs (Fig. 5C,D). As a result, the genotypes G3 and G5 were the best; they were found near the origin and had the lowest PCs values (Fig. 2C). Genotypes G5 and G1 showed practically zero scores for both PC1 and PC2, indicating that these genotypes were less interacting with the locations, according to the AMMI2 biplot (Fig. 5D) graphical analysis for PC1 and PC2. Our findings are in line with those of Gauch and Zobel^78^ and Khan^69^, who discovered that the first two PCAs are enough for the AMMI model's projection, despite some research suggesting the use of the first four PCs in the multi-environment trial^79^. AMMI biplots were used in several research, such as those on barley^80^, rice^25^, sorghum^42^, and maize^74^, to identify the corresponding genotypes.

The average environment coordinate (AEC) technique was used to assess trait performance and genotype stability^46^. The average environment coordinate is the line that crosses the perpendicular line of the biplot origin. The direction in which the arrows point to a tiny circle represents the genotype mean yield^81^. In the meanwhile, genotype stability is shown by the perpendicular lines that cross the biplot origin^82^. Higher genotype stability is seen in genotypes that are nearer the AEC line^83^. The optimal genotype is stable and has a high mean yield per hectare. G3 and G5 genotypes were associated with a high mean yield per hectare (Fig. 5A). While G5 was unstable, as seen by longer lines from AEC, genotype G3 performed steadily with lines closer to AEC. While G1 was shown to be more closely connected to AEC lines, suggesting consistent performance and a better mean yield per ha production, Fig. 5B revealed that genotypes with a higher mean yield per ha were G3. The genotypes positioned in the inner circle were considerably more appropriate than those in the outside circle. The genotypes that are next to and closer to the inner circle are thought to be best; nevertheless, in a few cases, no genotype was discovered within the inner circle^69,84^. Figures 7A,B show the genotype ranking according to mean and stability routine. The AEC approaches were used in GGE biplot analysis to estimate genotype yield and stability^46^. The average environment coordinate (AEC) is the line that goes through the biplot origin. It is determined by averaging the PC1 (mean yield) and PC2 (stability) scores for every environment^45^. A greater mean yield is shown by being closer to the concentric circle. The best genotypes for selection are those that have a high mean yield and good stability. They have the smaller vector from the AEC and are situated near the origin in the biplot. The genotypes with yield performance larger than the mean yield are on the right side of the line without an arrow, whereas the genotypes with yield performance less than the mean yield are on the left side of this line. The most stable and highly productive genotypes in this investigation were G3 in both treatments (Fig. 6A,B). Each environment's rating is shown by how close the environmental point is to the ideal point (small arrow) in Fig. 7 (environment rank). Ruswandi^85^ states that the perfect environment is the one that is closest to the optimum point. The location that is closest to the ideal point, E2, was the environment that was outside the circle and had a sufficiently wide angle with the ideal point in our study of the AWD. This was followed by E7 and E1 (As seen in Fig. 7A). This suggests that although other settings may be utilized to select adaptable genotypes, the E2 was the most suitable environment for selecting stable and great-producing genotypes. In Fig. 7B, the E9 sites are the most wanted because they are the closest to the ideal environment, while the E7 locations were thought to be the most undesired.

According to Azam et al.^86^ and Hossain et al.^87^ the what-won-where perspective of the GGE bi-plot is the best model for multi-environment trial data. It enables you to classify the environments and identify the genotype that performs best in each. This biplot piqued the interest of many academics since it presents key ideas including crossover GE, massive environment difference, and specialized adaptation in a visual manner. To include all additional genotypes, a polygon is initially created on the genotypes that are furthest from the biplot origin. Beginning at the biplot origin, draw the perpendicular lines to each side of the polygon^88^. In the first mega-environment with AWD, Vertex genotype G3 emerged as the dominant genotype. However, in the second mega-environment, genotype G5 emerged victorious (Fig. 4A). In the mega-environment of CF therapy, genotype G3 fared better as well (Fig. 4B). "Different cultivars should be selected and deployed for each different environment," the conclusion states. The rice workers, namely Akter^82^, Mohan^89^, and Siddi^90^, reported similar findings.

The MGIDI is a relatively new technique that is being used in an increasing number of crops to assist in discovering a better genotype for many characteristics simultaneously^86,87,91,92^. The Euclidean genotype-ideotype distance is computed using a factor analysis scores^93^. The MTSI is based on the projection of factor analysis results onto the genotype-ideotype distance. Plant breeders may find the MGIDI beneficial in choosing genotypes based on numerous characteristics since it offers a robust and understandable selection strategy. Based on the characteristics under evaluation, the genotypes with lower MGIDI values have stronger stability. Six characteristics were taken into account for MGIDI analysis in our investigation: G3 genotypes were thought to be the most desirable or stable. The MGIDI index is a unique and easy-to-interpret selection procedure that has many practical applications to obtain long-term genetic gain in primary traits (such as grain yield) without jeopardizing gains of secondary traits (such as plant height). The proportion explained by each factor towards the MGIDI index, i.e., “strengths and weaknesses view” (Fig. 10A,B), is an important graphical tool to identify the strengths and weaknesses of test hybrids in terms of “trait (group of traits) need to be improved” and is an added advantage over existing indices. A similar study was reported by Olivoto and Nardino^41^, who evaluated a set of 13 strawberry cultivars, wherein the strengths were described by employing MGIDI.

## Methods

### Description of the site and soil

**Figure 11:**
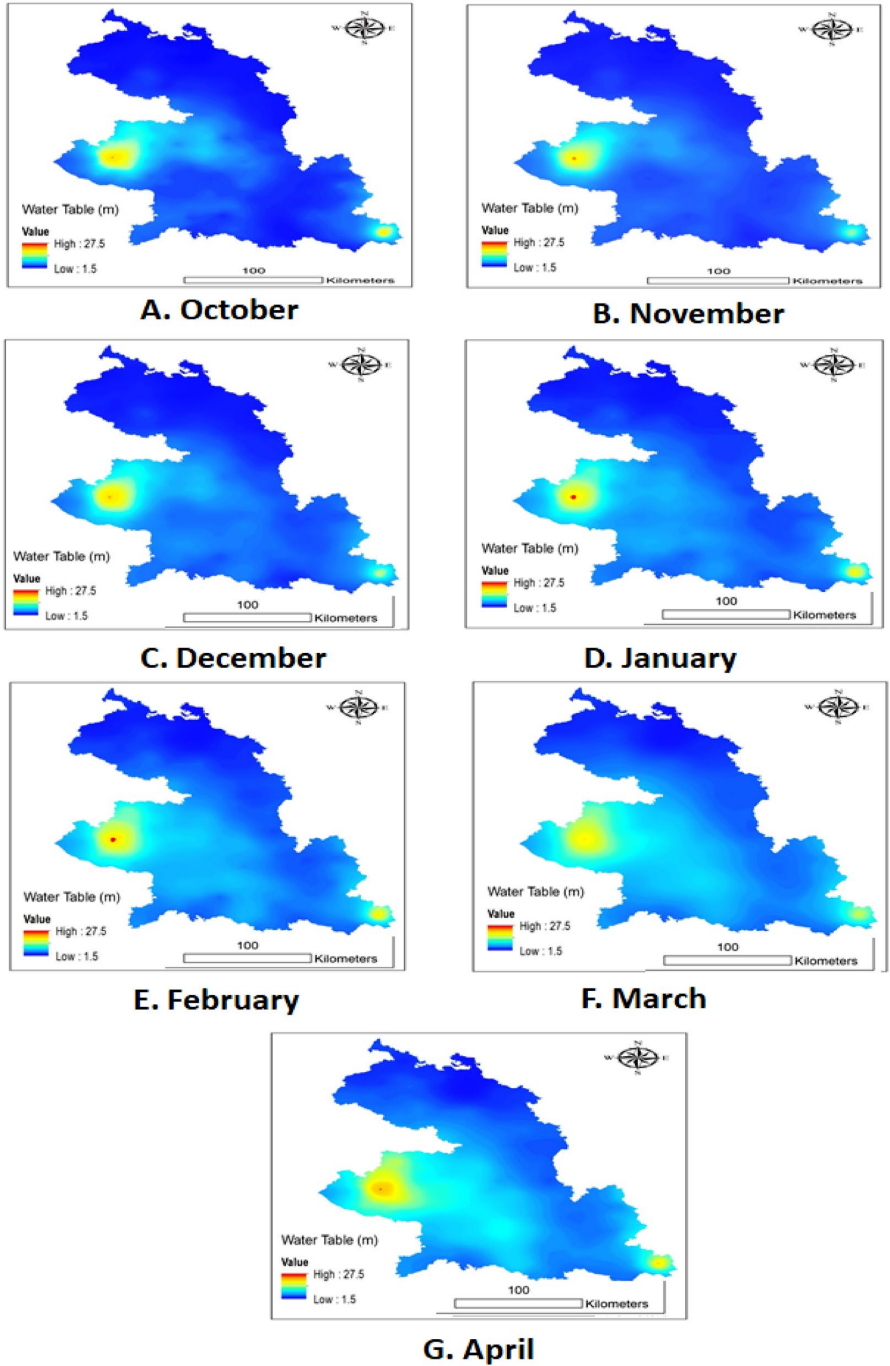
Water status of soil from October to April in Bangladesh. The data were collected from 2010 to 2019 by using the web-based warehouse inventory search tool (WIST).

In Bangladesh, the Boro season lowers the water table and calls for greater irrigation. The shortage of water in Bangladesh's western Borendra area makes rice production more difficult. The ideal sites for this experiment were identified using surface reflectance (SR) data obtained from the Moderate Resolution Imaging Spectra Radiometer (MODIS) satellite equipped with a TERRA sensor and a product called MOD09. A sinusoidal grid with a horizontal resolution of 250 m is used to protect all of the data. The data was collected using the web-based warehouse inventory search tool WIST between 2010 and 2019. Month-by-month maps with the relevant photos were created when the data analysis was finished (Table 7 and Fig. 11).

**Table 7:** List of the locations selected for the experiment location detail during October 2019 to April 2020. (*Source*: Bangladesh Meteorological Department (BMD), Rajshahi region).

Name locations	Environment code	AEZ (Agro-ecological zone)
Nachole	E1	High Ganges River floodplain
Talando	E2	Active Ganges floodplain
Kolma	E3	Active Ganges floodplain
Kismatgarkor	E4	Active Ganges floodplain
Dhuroil	E5	Active Ganges floodplain
Shavoadanga	E6	Active Ganges floodplain
Biprabelgharia	E7	High Ganges River floodplain
Parsodanga	E8	Active Ganges floodplain
Gobindasi	E9	Madhupur tract

**Figure 12:**
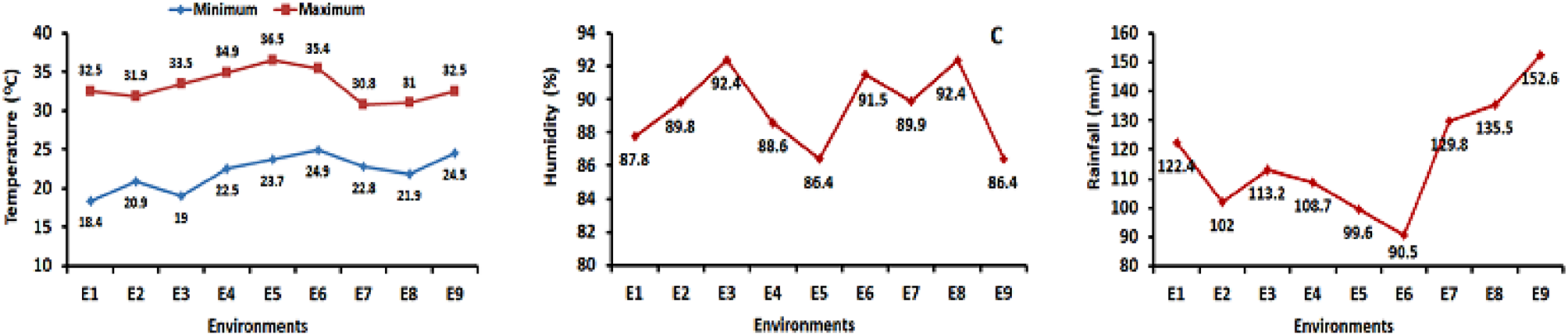
Temperature, humidity, and rainfall variation during grain filling period in both AWD and CF methods at 9 environments in the rice crop season of 2019–2020.

The High Barind Tract (HBT), which covers an area of around 1600 km^2^ (159,964 hectares), is located in Bangladesh's North-West Rajshahi Division at coordinates of 24.40–24.80° N and 88.30–88.80° E. Its terraced terrain, poor fertility soil, sparse vegetation, lack of main river routes, and relatively low rainfall combined with a protracted dry season from October to May set it apart from other regions of the nation^94^. Because of its high summertime air temperatures, which can reach maximums of 35–40 °C in April and May, its limited groundwater reserves and recharge, the poor water-holding capacity of surface soil during the post-rainy season, and its relatively low and irregular precipitation (average annual rainfall of 1440 mm, which can vary spatially and seasonally between 790 and 2200 mm), it is known as the most drought-prone region in the country (Fig. 12). Unlike Bangladesh's floodplain regions, which experience yearly flooding, the HBT is comparatively elevated (9–47 m above sea level^94^.

The High Barind Tract's soils reacted with a mild acidity. According to the USDA classification, they were of medium- to heavy texture, with textural grades of silt loam for six soils, silty clay loam for three soils, and silty clay for the remaining soil. The majority of soils have significant clay contents, ranging from 16.0 to 43.9%, with topography being the primary cause of the difference. Quartz predominated in the 2–20 gm silt fraction, with minor amounts of mica, plagioclase, K–feldspar, and chlorite^95^. Generally speaking, the texture of the soil was clay to silty clay underneath and silty clay to clay on top. In every profile, there was a gradual rise in the amount of clay from the surface down^96^.

### Plant components and the layout of field experiments

The present investigation used AWD and CF systems, whereby the five genotypes including the genetic materials better than the others (Table 8). Table 8 displays the genotype name, code, collection source, and varietal description. The field experiment was performed from January to May 2020, throughout the Boro season. 30 days after the seedlings were grown into the seedbed, they were moved per hill, with a 20 cm × 20 cm gap between rows and plants^97^. Plants were spaced 20 cm apart from one another in this experiment, and rows were spaced 20 cm apart. The nine settings in the multi-environment trials were performed under alternating cultivations of early and standard planting seasons. Rice cultivars and irrigation schedules were the two variables combined, and they were set up in a split-plot design with three replications. The main plot was the irrigation regime, while the subplot was the rice genotype. Rice cultivars were assigned at random to three replications at a subplot. A 6-m buffer was placed between the two irrigation regime plots to prevent sub flow from the continuous flooding plot from contaminating the plot. The experiment used a split-plot design with three (3) replications, maintaining a unit plot size of 8 × 5 m. To keep the soil fertility in the experimental units at the optimal level for rice cultivation in Bangladesh, additional chemical fertilizers were sprayed at rates of 220–120-90–60 kg/ha, respectively, including urea, triple super phosphate, muriate of potash, and gypsum (CaSO_4_.2H_2_O)^98^. The full doses of TSP and muriate of potash, along with half of the dose of urea, were applied as the basal fertilizer application, and the remaining 50% of the nitrogenous fertilizer was divided into two halves. The first one was used during tillering, and the second one was used during booting. All crucial intercultural tasks, such as gap filling, routine weeding, applying insecticides, adding fertilizer to the top, and so on, were completed throughout the growing season to support the plants' healthy growth and development. Experimental research and field studies on rice including the collection of rice seeds comply with our institutional, national, and international guidelines and legislation.

**Table 8:** List of the Genotypes selected for this experiment.

Genotype name	Genotype code	Source	Variety description
Binadhan-10	G1	BINA	Pedigree: IR64197-3B-14-2*, Grain type: Medium slender, Potential yield: 5.5–6.0 t ha^-1^, Duration: 125–130 days
Binadhan-8	G2	BINA	Pedigree: IR66946-3R-1-1*, Grain type: Medium bold, Potential yield: 5.0–7.0 t ha^-1^, Duration: 130–135 days
BRRI dhan29	G3	BRRI	Parentage: BG90-2/BR 46-51-5, Grain type: Medium slender, Potential yield: 7.50 t ha^-1^, Duration 150–160 days
BRRI dhan47	G4	BRRI	Parentage: IR515111-B-B-34-B*/TCCP266-2-49-B-B-3*, Grain type: Medium bold, Potential yield: 6.0 t ha^-1^, Duration: 145–152 days
Binadhan-17	G5	BINA	Pedigree: (SAGC-7 (GSR)*), Grain type: Medium bold, Potential yield: 7.50 t ha^-1^, Requires fewer inputs, Saves 30% water, GSR variety, Duration: 113–118 days

**Figure 13:**
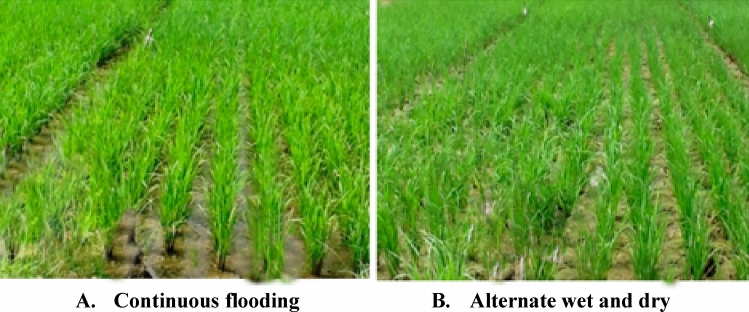
Comparison of canopy of crop and soil wetness status under two different irrigation regimes i.e., continuous flooding and alternate wet and dry conditions during the rice tillering stage at Nachole during Boro season. (**A**) Continuous flooding, (**B**) Alternate wet and dry.

Two AWD irrigation regimes were used as a water-saving strategy in place of constant flooding. Throughout the whole cropping season, under the continuous flooding regime, a constant water layer of 5 to 10 cm was maintained, depending on the crop growth stage (Fig. 13a). Irrigation was the first water-saving measure considered when the soil matric potential (AWD30) reached 30 kPa (Fig. 13b). The average daily soil matric potential (SMP) was measured and hourly monitored using Watermark Granular Matrix sensors (WGMs; model: EC06-783, Irrometer, Co., Riverside, CA, USA). WGMs provide an indirect way to quantify SMP by directly measuring soil water tension. Two WGMs were placed into each plot at a depth of 15 cm to lessen the variation in soil water content caused by field leveling. As soon as the standing water level hit 20 mm, irrigation was turned on while using the continuous flooding irrigation technique. Thirty millimeters of irrigation water were applied to the rice during its vegetative growth stage, and eighty millimeters were applied during its reproductive phase. During the rice vegetative stage, 30 mm of irrigation water was applied at each irrigation event under the AWD treatments (AWD30). During the rice reproductive phase, irrigation was similarly managed under AWD treatments to continuous flooding. This was done to avoid or reduce sterility. Before imposing irrigation regimes on the treatments, 100 mm of irrigation water was administered for paddling and transplant survival^99^.

### Data gathering

In each plot, 10 randomly selected plants were taken for sampling all of the phenotypic data. Plants were harvested when 85% of the grains of the panicles turned a golden yellow color. Three biological replicates (10 plants per replicate for each trait) were used to collect data on growth and yield-related traits. The studied days to maturity (DM) trait was measured from the sowing to harvesting day. At harvest, plant height (PH) was determined from the soil surface to the highest part of the plant; Effective tiller (ET) was counted as the number of panicles bearing tillers per hill; Panicle length (PL) was determined from the bottom of the peduncle to the topmost of the panicle; Thousand seeds weight was (TSW) recorded by counting one thousand seeds manually from each pot and weighed using an electronic balance. The grain yield was obtained using a crop cutting test (CCT) of 1 m by 1 m areas of each experimental plot. The harvested grains were dried, winnowed, and weighed. The weight was then converted to per unit area crop yield based on 14% grain moisture content and presented as grain yield (t/ha). Tons per hectare were calculated from the seed yield of each plot.

### Statistical analysis

The average data of various traits (Supplimentary file 1 and 2) were analyzed statistically^100^ and biometrically^101,102^. R version 4.2.1was used for all statistical analysis and data visualization^103^. The analysis of variance, AMMI analysis of variance^104^, genotype plus genotype × environment (GGE) biplot^46^, and stability statistical analysis were all performed using the "metan" R Package^93^. The AMMI model uses two techniques: multiplicative interaction effect and additive main effects of environments and genotypes, as well as ANOVA and singular value decomposition^105^. A genotype main effect plus genotype-by-environment interaction (GGE) biplot was used to display the winning genotypes and mega-environments (MEs) for yield-related features and grain production^46,106^. Using a "which-won-where" biplot, investigations on MEs and the optimal genotype in each habitat were carried out. "Discriminativeness vs. representativeness" biplots were used to assess the ideal conditions for each characteristic. Ideal genotypes exhibiting high mean performance and stability across all parameters were shown using "ranking genotypes" biplots. It assigns a score to genotypes according to many attributes, using the multi-trait genotype-ideological distance index (MGIDI) proposed by Olivoto41. The statistical analysis for the multi-trait genotype–ideotype distance index (MGIDI) was performed using the R Package "metan" version 1.16.0.

Different stability statistics such as ASV, HMRPGV, Wricke's ecovalence, and Thennarasu's statistics were calculated by the following method which is described below:-

### *AMMI* stability value (ASV)

Purchase^107^ developed another stability statistic based on the two first IPCA scores for each genotype. The AMMI stability value (ASV) is the distance from the coordinate point to the origin in a two-dimensional scattergram of IPCA1 scores against IPCA2 scores. This measurement is described as follows:

$$\text{ASV}=\text{ASV}\sqrt{{\left(\frac{{\text{SSIP}}_{1}}{{\text{SSIP}}_{2}}\times {\text{PC}}_{1}\right)}^{2}+{\left({\text{PC}}_{2}\right)}^{2}}$$

where SSIP1/SSIP2 is the ratio between the sum of squares from the first and second interaction principal component axis, and PC1 and PC2 are the genotypic scores of these components in the AMMI model. The genotype with the lowest value of this statistic would be more stable.

Wricke’s Ecovalence (W2) Wricke^108^ proposed the concept of ecovalence as the contribution of each genotype to the GEI sum of squares.

$${W}_{i}^{2}=\sum \left({X}_{ij}-{\overline{X} }_{i.}-{\overline{X} }_{.j}+{\overline{X} }_{..}\right)$$

where Xij is the grain yield of genotype ith in environment jth; Xi. is the mean grain yield of genotype ith; X.j is the mean grain yield of the environment jth; and X. is the grand mean.

The harmonic mean of RPGV (HMRPGV) is the third BLUP-based stability parameter that considers stability, adaptability, and mean performance simultaneously. This parameter is calculated as follows:

$${HMRPGV}_{i}=\frac{n}{\sum_{j=1}^{n}\left(\frac{n}{{RPGV}_{ij}}\right)}$$

In the above formulas, GVij, _j, and E are the genotypic values (BLUP) for the ith genotype in the jth environment, the grand mean for each environment j, and the number of environments, respectively. As has been mentioned by Resende^109^, the highest values of these parameters are suitable.

### Thennarasu’s statistics

Since the rank of genotypes in the specific environments cannot be done according to the phenotypic values, the stability of test genotypes has to be estimated independently of the genotypic effect. To solve this challenge, a correction of ranking patterns of test genotypes and environments based on the corrected phenotypic values was developed. Four NP^(1–4)^ statistics are a set of alternative non-parametric stability statistics defined by Thennarasu^110^. Indeed, these parameters are based on the ranks of adjusted means of the genotypes in each environment.

## Conclusions

Considering the impact of global warming, it is imperative to incorporate effective irrigation methods in rice cultivation that not only consider the water requirements of plants but also prioritize factors such as WUE, physiological processes, and resilience in stressful situations. The performances of each genotype in all situations for the AWD technique and the CF system were found to be noteworthy, it is possible to conclude that all genotypes were comparable to one another. Binadhan-10 exhibits the lowest G × E interaction in the AWD technique, whereas BRRI dahn29 performed better and exhibited less G × E interaction in both the CF system and the AWD method, according to the adaption map and AMMI biplot stability. It also offered a respectable understanding of how well rice genotypes respond to a variety of settings using stability analysis. Yield stability varies throughout genotypes in different settings. The AMMI statistical model has proven to be a useful tool for determining the appropriate genotypes for a given environment, as well as for varied settings. The most responding genotypes in the CF system were Binadhan-17, BRRI dhan47, and Binadhan-8 in the AWD approach. The genotypes that were evaluated revealed that Binadhan-10 had a more stable genotype for the AWD system and that BRRI dhan47 & BRRI dhan29 were the best fits for all the environments in both systems. Two rice varieties, Binadhan-10 and BRRI Dhan47, may be deployed and shown at the farmer level in that area of fields for sustainable food security and smart farming. AWD has also been found to be a delicate and effective irrigation method that does not compromise grain production and quality, while also being economically feasible.

### Supplementary Information

Supplementary Information 1.

Supplementary Information 2.

## Data Availability

This published manuscript and its accompanying information files contain all of the data created or analyzed during this investigation.
